# Identifying Core Functions of an Evidence-Based Intervention to Improve Cancer Care Quality in Rural Hospitals

**DOI:** 10.3389/frhs.2022.891574

**Published:** 2022-04-28

**Authors:** Madison M. Wahlen, Mary C. Schroeder, Erin C. Johnson, Ingrid M. Lizarraga, Jacklyn M. Engelbart, David J. Tatman, Cheyenne Wagi, Mary E. Charlton, Sarah A. Birken

**Affiliations:** 1 Department of Epidemiology, University of Iowa, Iowa City, IA, United States,; 2 Department of Pharmacy Practice and Science, University of Iowa, Iowa City, IA, United States,; 3 Department of Management and Entrepreneurship, University of Iowa, Iowa City, IA, United States,; 4 Department of Surgery, University of Iowa Hospitals and Clinics, Iowa City, IA, United States,; 5 Department of Implementation Science, Wake Forest University, Winston-Salem, NC, United States

**Keywords:** core functions, guideline-concordant care, rural cancer disparities, barriers to quality cancer care, rural healthcare access, evidence-based intervention, adaptation, quality improvement

## Abstract

**Background:**

Rural patients experience worse cancer survival outcomes than urban patients despite similar incidence rates, due in part to significant barriers to accessing quality cancer care. Community hospitals in non-metropolitan/rural areas play a crucial role in providing care to patients who desire and are able to receive care locally. However, rural community hospitals typically face challenges to providing comprehensive care due to lack of resources. The University of Kentucky’s Markey Cancer Center Affiliate Network (MCCAN) is an effective complex, multi-level intervention, improving cancer care in rural/under-resourced hospitals by supporting them in achieving American College of Surgeons Commission on Cancer (CoC) standards. With the long-term goal of adapting MCCAN for other rural contexts, we aimed to identify MCCAN’s core functions (i.e., the components key to the intervention’s effectiveness/implementation) using theory-driven qualitative data research methods.

**Methods:**

We conducted eight semi-structured virtual interviews with administrators, coordinators, clinicians, and certified tumor registrars from five MCCAN affiliate hospitals that were not CoC-accredited prior to joining MCCAN. Study team members coded interview transcripts and identified themes related to how MCCAN engaged affiliate sites in improving care quality (intervention functions) and implementing CoC standards (implementation functions) and analyzed themes to identify core functions. We then mapped core functions onto existing theories of change and presented the functions to MCCAN leadership to confirm validity and completeness of the functions.

**Results:**

Intervention core functions included: providing expertise and templates for achieving accreditation, establishing a culture of quality-improvement among affiliates, and fostering a shared goal of quality care. Implementation core functions included: fostering a sense of community and partnership, building trust between affiliates and Markey, providing information and resources to increase feasibility and acceptability of meeting CoC standards, and mentoring and empowering administrators and clinicians to champion implementation.

**Conclusion:**

The MCCAN intervention presents a more equitable strategy of extending the resources and expertise of large cancer centers to assist smaller community hospitals in achieving evidence-based standards for cancer care. Using rigorous qualitative methods, we distilled this intervention into its core functions, positioning us (and others) to adapt the MCCAN intervention to address cancer disparities in other rural contexts.

## INTRODUCTION

Rural cancer patients experience significant barriers to accessing cancer care; primarily travel-related concerns including transportation, lodging, and financial burdens (1). While some patients choose to travel to large urban cancer centers for treatment, many decide to stay close to home. Further, some do not have the resources to travel, are too ill to benefit from aggressive care, or prefer to receive care locally. Therefore, community hospitals in non-metropolitan and rural areas play a critical role in providing care to many patients who otherwise would not have access. Despite their importance, rural hospitals typically face challenges to providing comprehensive care such as lack of resources, staff shortages, lack of specialist providers, and high-risk patient populations (2–4). They also often lack the infrastructure to collect extensive data on their cancer patients to monitor quality of care and identify processes in need of improvement, which can contribute to guideline-disconcordant care and poorer outcomes than might be received at non-rural hospitals (5, 6). Supporting these rural hospitals in developing the infrastructure to monitor their quality of care and develop programs and services to meet patient needs could improve the standard of care for rural cancer patients and aid rural cancer programs in maintaining the revenue they need to remain viable.

The University of Kentucky Markey Cancer Center Affiliate Network (MCCAN) is a complex, multi-level intervention aimed at improving cancer care in community hospitals, including those in rural and underserved areas. Several other leading cancer centers have partnered with rural hospitals to address rural cancer disparities; however, MCCAN is among the first to demonstrate significant improvement in guideline-concordant treatment metrics associated with improved survival outcomes (7). To support efforts related to data collection and monitoring, quality assessment and improvement, and cancer program development and goal setting, MCCAN uses the American College of Surgeons Commission on Cancer (CoC) evidence-based standards of cancer care. CoC is “a consortium of professional organizations dedicated to improving survival and quality of life for cancer patients through standard setting, which promotes cancer prevention, research, education, and monitoring of comprehensive quality care” (8). MCCAN helps hospitals that have never been CoC accredited build the infrastructure and navigate the process, and assists those that are already CoC accredited with maintaining accreditation and conducting targeted quality improvement efforts, among other support activities (7).

Given MCCAN’s success, it serves as a promising model for improving access to high quality cancer care in other rural settings, but it has not yet been expanded beyond its intended context. Other rural states and geographic areas could benefit from an approach similar to those of MCCAN as well. For example, the largely rural state of Iowa is an ideal setting in which to pilot the scale up of an adapted MCCAN model based on the many similarities between the two states. National Cancer Institute (NCI)-designated University of Iowa Holden Comprehensive Cancer Center and several rural community hospitals across Iowa seek to adapt MCCAN to support Iowa community hospitals in achieving CoC quality standards. Given MCCAN’s success and a desire to extend its benefits beyond Kentucky, there is a need to understand how to implement MCCAN in other settings and determine the mechanisms of its effectiveness.

In practice, interventions are often adapted to facilitate implementation in reaction to poor fit in new contexts or populations (9). In many cases, this kind of reactive adaptation improves interventions’ implementation but compromises their effectiveness (10). In order to adapt MCCAN to facilitate its implementation in Iowa while preserving previously demonstrated levels of effectiveness in improving care processes and outcomes (7), it is necessary to identify MCCAN’s core functions— i.e., the components of an intervention that makes it effective, and distinguish these from its forms – i.e., the activities necessary to carry out the core functions which can be adapted to satisfy demands of alternative contexts (11, 12). Scholars have successfully identified core functions underlying an increasing number of interventions, including an intervention to facilitate hospice referrals (13), as well as Water, Sanitation, and Hygiene (WaSH) programs, and the Accountability for Cancer Care through Undoing Racism and Equity (ACCURE) intervention, an intervention intended to address racial disparities in completion of cancer treatment, not yet published work.

While MCCAN has demonstrated its ability to improve cancer care by partnering with and supporting rural affiliate hospitals in achieving the evidence-based quality standards set forth by the CoC (7), the mechanisms underlying MCCAN’s effectiveness are unknown. Our objective was to identify MCCAN’s core functions as part of a broader NCI-funded study with a long-term goal of adapting MCCAN for implementation in rural settings beyond Kentucky to increase access to high-quality cancer care and mitigate rural cancer survival disparities.

## MATERIALS AND METHODS

### Overview

To identify MCCAN core functions (the components of an intervention that makes it effective), we used the methods set forth by Kirk et al. with guidance from author SAB, who helped develop the methods (12). Since MCCAN has not been rigorously defined previously, we reviewed the existing materials through meetings with MCCAN leaders involved in overseeing and supporting affiliate hospitals in the intervention to create a document describing MCCAN’s forms (the activities necessary to carry out the core functions which can be adapted to satisfy demands of alternative contexts) from their perspective. We then interviewed representatives from five MCCAN affiliate hospitals with the objective of distilling MCCAN leader-identified forms into MCCAN core functions.

This study and materials (i.e., interview guide, recruitment emails) were approved by the University of Iowa IRB.

### Developing a Document Describing MCCAN’s Forms

A research assistant (CW) compiled a document describing MCCAN’s forms using existing documents that included descriptions of MCCAN, including internal documents (i.e., a flier about MCCAN, a table describing MCCAN staff roles), and published characterizations of the intervention (7, 14). CW compiled for inclusion in the document notes on 1) the purpose and role of each aspect of the intervention, 2) the aspects of the intervention that were essential to its success, 3) the purpose of the intervention, and 4) methods and conditions necessary to implement the intervention. The study team reviewed the document for accuracy and clarity. We then incorporated feedback on the document from MCCAN’s medical director, administrative director, quality director, and certified tumor registrar (CTR). The final document (Supplementary Table 1) defined MCCAN’s forms.

## IDENTIFYING MCCAN CORE FUNCTIONS

### Sampling and Recruitment

Increasing the number of rural hospitals that can achieve CoC accreditation is one of the primary goals of the University of Iowa research team and their community hospital partners. As such, we recruited representatives from eligible MCCAN affiliate hospitals because they are key stakeholders in the implementation process, understand how MCCAN works, and have gone through the accreditation process at various stages. We targeted representatives from MCCAN affiliate hospitals for inclusion if the hospital was not CoC-accredited prior to joining MCCAN and became accredited within 3 years of joining. Of the five Kentucky community hospitals that met eligibility criteria, four are located in rural areas (as defined by the Rural-Urban Continuum Codes classification scheme) and one is in an urban area but serves a rural population.

Based on input from MCCAN’s medical and administrative directors, we recruited both clinicians and administrators (i.e., program coordinators) to participate because of their varied perspectives and roles as influencers of change at their facilities. MCCAN’s administrative director invited 11 representatives across the five hospitals to participate via email. After the initial contact, a research coordinator emailed representatives to schedule the interview. We offered participants a $50 check to incentivize their participation. Our final sample included eight representatives (three clinicians, four administrators, and one certified tumor registrar) from the five eligible hospitals.

### Data Collection

The interview guide (Supplementary Table 2) was developed following Kirk et al.’s method of identifying core functions (12). We then piloted the interview guide with Markey’s CTR who previously worked at a MCCAN affiliate hospital. This pilot interview was not included in analyses. Two members of the research team with qualitative and clinical expertise (ECJ, JME) conducted semi-structured virtual interviews via Zoom with representatives between June 29th through August 12th, 2021. The document identifying MCCAN’s forms was available to participants at the time of interview for clarification about the MCCAN intervention if necessary. Interviews lasted between 39 and 72 min (mean 48.25 min). The interviews were audio-recorded and transcribed verbatim using the web-based service Rev.com. We deidentified transcripts for analysis.

### Data Analysis

We developed an initial codebook prior to coding based on the Model for Adaptation Design and Impact (10). The final version of the codebook contained a priori codes based on the interview guide (i.e., what drives MCCAN’s success) and some inductively identified codes. To refine the codebook and the reliability of our interpretation of the data, four study team members (MMW, MCS, EMJ, SAB) iteratively co-coded subsets of three transcripts, discussing discrepancies in application of the codes and reconciling differences. Once we reached consensus and refined the codebook, a research assistant (MMW) coded the remaining transcripts.

We generated reports for each code and MMW identified themes related to how MCCAN engaged affiliate sites in implementing CoC standards and improving care quality according to guidelines discussed among the study team. Themes were written to be comprehensive of interview excerpts and include a subject and verb (i.e., “MCCAN provides resources to affiliate hospitals”). To improve reliability in coding, four team members (MMW, MCS, ECJ, SAB) discussed the themes and resolved discrepancies in interpretation. MMW, with guidance from SAB, analyzed the final list of themes for codes encompassing MCCAN strategies to inform the core functions. Core functions *of the intervention* were defined as features of the intervention that were necessary and collectively sufficient to achieve the effectiveness outcome of improved cancer care. Core functions *of the implementation* were defined as features of the intervention that were necessary and collectively sufficient to achieve the implementation outcomes [i.e., feasibility of achieving CoC standards (15)]. We then presented the core functions that we identified to MCCAN leadership (medical director, administrative director, and quality director) for confirmation of face validity and comprehensiveness of the functions. Based on their feedback that some of the core functions were redundant, we consolidated the original list of core functions.

Finally, as an additional metric of validity to increase our confidence in the interpretation of the results, we identified a related theory of change from the literature to map core functions onto. This step is preferred according to Kirk et al.’s methods (12), as MCCAN was not theory-based in its creation. SAB used her in-depth knowledge of cancer care delivery and implementation theories to identify mechanisms of change underlying each set of core functions: intervention and implementation.

## RESULTS

### Interviews

We conducted a total of eight interviews with participants from the five eligible MCCAN affiliate hospitals. From a single institution, the greatest number of participants was three, while the fewest was one participant. The hospital and cancer program roles of each participant are described in Table 1.

### Intervention Core Functions

**FIGURE 1 |:**
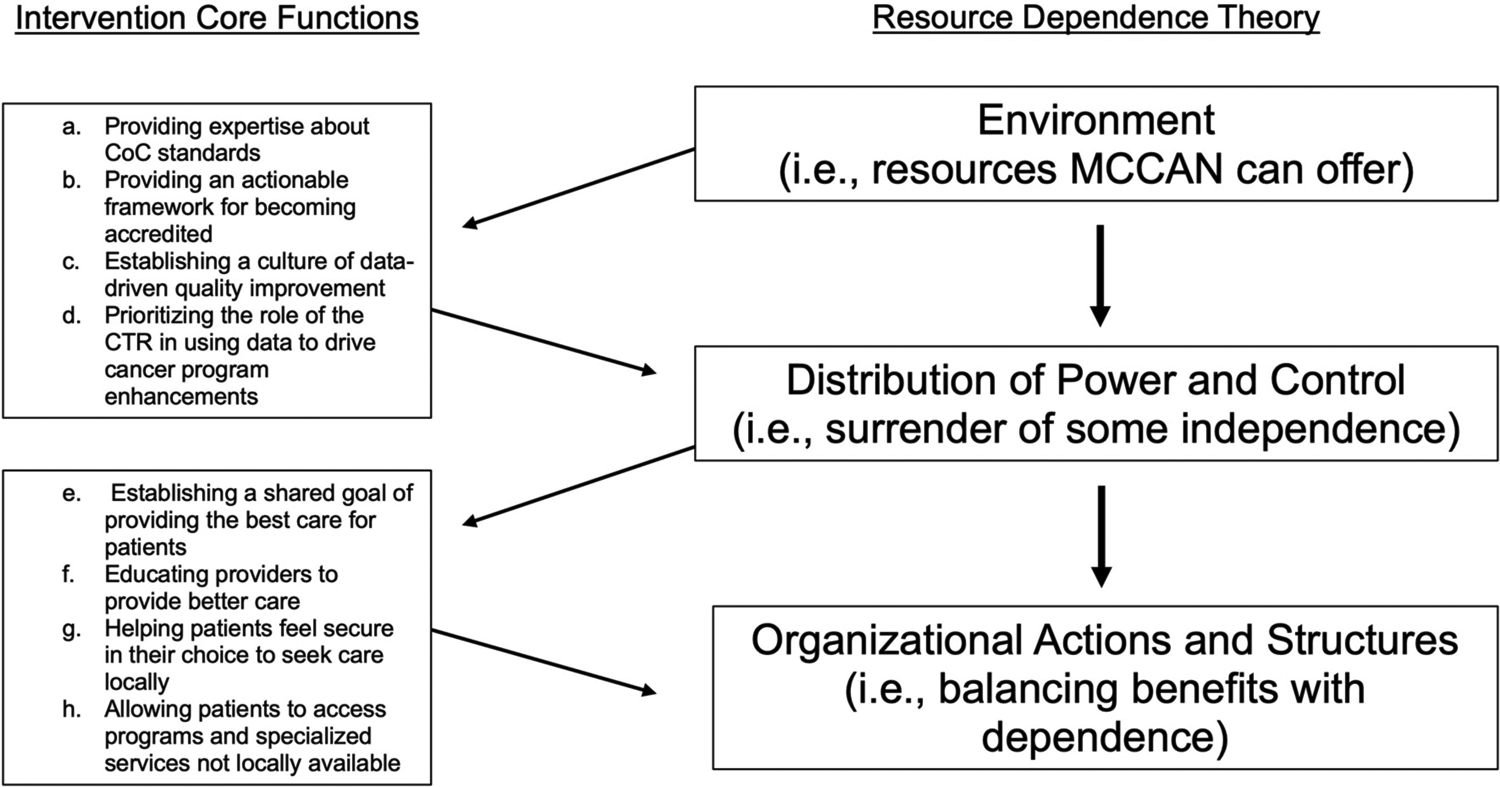
Intervention core functions mapped onto resource dependence theory of change [18].

We identified eight intervention core functions as necessary and collectively sufficient to improve care quality and describe them in detail below (Table 2; designated below as a-h). These core functions can be explained with Resource Dependence Theory (RDT) (Figure 1; i.e., theory of change) (16). Applied to MCCAN, RDT suggests that affiliate hospitals improve quality when they balance the benefits of affiliation with MCCAN against the dependence that comes with affiliation.

### Environment and Dependence – Intervention Functions a-d

MCCAN provided the tools affiliates needed to adhere to CoC standards of care and improve care quality for patients at their facilities. These benefits of affiliation are demonstrated by the following functions: a) *providing expertise about CoC standards*, b) *providing an actionable framework for becoming accredited*, c) *establishing a culture of data-driven quality improvement, and* d) *prioritizing the role of the certified tumor registrar (CTR) in using data to drive cancer program enhancements*. In order to access these benefits, affiliates had to trade some level of independence by aligning with a broader network and complying with the MCCAN affiliate agreement and standards set forth by the CoC.

a) *Providing expertise about CoC standards and* b) *an actionable plan and framework for becoming accredited*. Information about CoC standards includes what CoC standards are, how they are interpreted, and how affiliates can demonstrate their achievement in ways that are acceptable to the CoC. For previously unaccredited institutions with varying levels of familiarity with the CoC, MCCAN served as the primary resource for understanding the expectations of the CoC and identifying the gaps and process modifications necessary to fulfill CoC standards and “guide[d affiliates toward]… the vision of the CoC” (participant [P] 4.1 [institution ID.individual ID]). In addition to information about accreditation standards, MCCAN provided a roadmap to give cancer programs the proper “stepping stones” (P4.3) to achieve those standards and established new programs, and provided ongoing support and guidance to sustain those programs. For example, MCCAN helped affiliates develop survivorship clinics, navigation services, and social work programs, all necessary components for accreditation, which have “really benefitted” patients and “would have never happened” without MCCAN’s guidance and the pursuit of CoC accreditation (P4.1).

c) *Establishing a culture of data-driven quality improvement and* d) *prioritizing the role of the CTR in using data to drive cancer program enhancements*. Data collection is a critical piece of accreditation. As part of the CoC accreditation process, CTRs in hospital cancer programs must collect demographic, tumor, treatment and outcome data on all cancer cases and submit it to the National Cancer Database (NCDB), which is used to compute the facility-level compliance of established cancer care quality measures as an accountability tool for quality assessment and improvement. MCCAN helped programs develop this data collection capability, provided guidance for the CTRs collecting the data, and supported cancer programs in interpreting the data and using it to plan quality improvement projects. In addition, MCCAN promoted maximizing the role of CTR to include roles beyond traditional data collection, such as preparing agendas and documenting minutes from cancer committee and tumor board meetings. Affiliates viewed the CTR role as “a vital role” (P1.2) that several programs did not staff prior to joining the affiliate network and would have faced major barriers to achieving accreditation without MCCAN’s assistance in helping to recruit and develop/train this position (Table 2d, P5.1). Affiliates valued access to data on their cancer cases and the CoC analytic tools, benchmarks and trend data, data but had difficulties in optimizing its collection and use without MCCAN’s infrastructure and support.

### Balancing Dependence With Benefits – Intervention Functions e-h

Organizational actions (i.e., joining MCCAN) depend on the balance between dependence on environmental features as represented by the core functions above, and the benefits afforded by the dependence on those resources (i.e., providing better care for their patients). Although affiliates surrendered some level of independence to access those resources, they ultimately balanced this with the benefits of e) *establishing a shared goal of providing the best care for patients*, f) *educating providers*, g) *helping patients feel secure in their choice to seek care locally*, and h) *allowing patients to access programs and specialized services not locally available*.

e) *Establishing a shared goal of providing the best cancer care for patients*, and f) *educating providers to help them provide better care, and helping patients make informed decisions about their care*. The shared pursuit of quality care for patients united MCCAN and affiliates as one community and facilitates trust within the network. One affiliate described the culture within the network as “familiness… we’re all one big thing here. We’re all trying to do the same thing. And I think that’s important” (P4.4). This shared goal was realized through keeping providers up-to-date on the latest developments in cancer care so that they can implement the information into their practice. MCCAN delivered this education through a variety of mechanisms including statewide affiliate conferences [i.e., “MCCAN held… conferences. [T]hese include education for the physician, physician assistants, nurses… and the patient navigator… cancer care pharmacist also” (P3.2)] and virtual meetings [i.e., “targeting a certain group of people… if it’s a subject about dietary… I can ask all of our dieticians to join … that 1 hour discussion at lunch” (P2.1–3.1)].

g) *Helping patients feel secure in their choice to seek care locally* and h) *allowing patients to access programs and specialized services not locally available*. The University of Kentucky is widely known and trusted across the state, and seeing their local institutions affiliated with the Markey Cancer Center assured patients that they would receive optimal care without having to travel large distances to the academic institution. One affiliate said, “I’ve had some people come and see me just specifically because I was a Markey affiliate. Like… ‘Hey, we want to stay. We’ve been in Markey. We want to have follow up with somebody who’s affiliated with them”‘ (P1.1). Patients were also assured access to all programs and services under the accepted standard of care. It is often difficult for under-resourced rural hospitals to directly provide all of the services (i.e., genetic counseling) needed to provide the best cancer care for their patients and to achieve accreditation, so MCCAN provided “an easier way of getting those services facilitated” (P1.2) through mechanisms such as telehealth or efficient referrals to Markey.

### Implementation Core Functions

**FIGURE 2 |:**
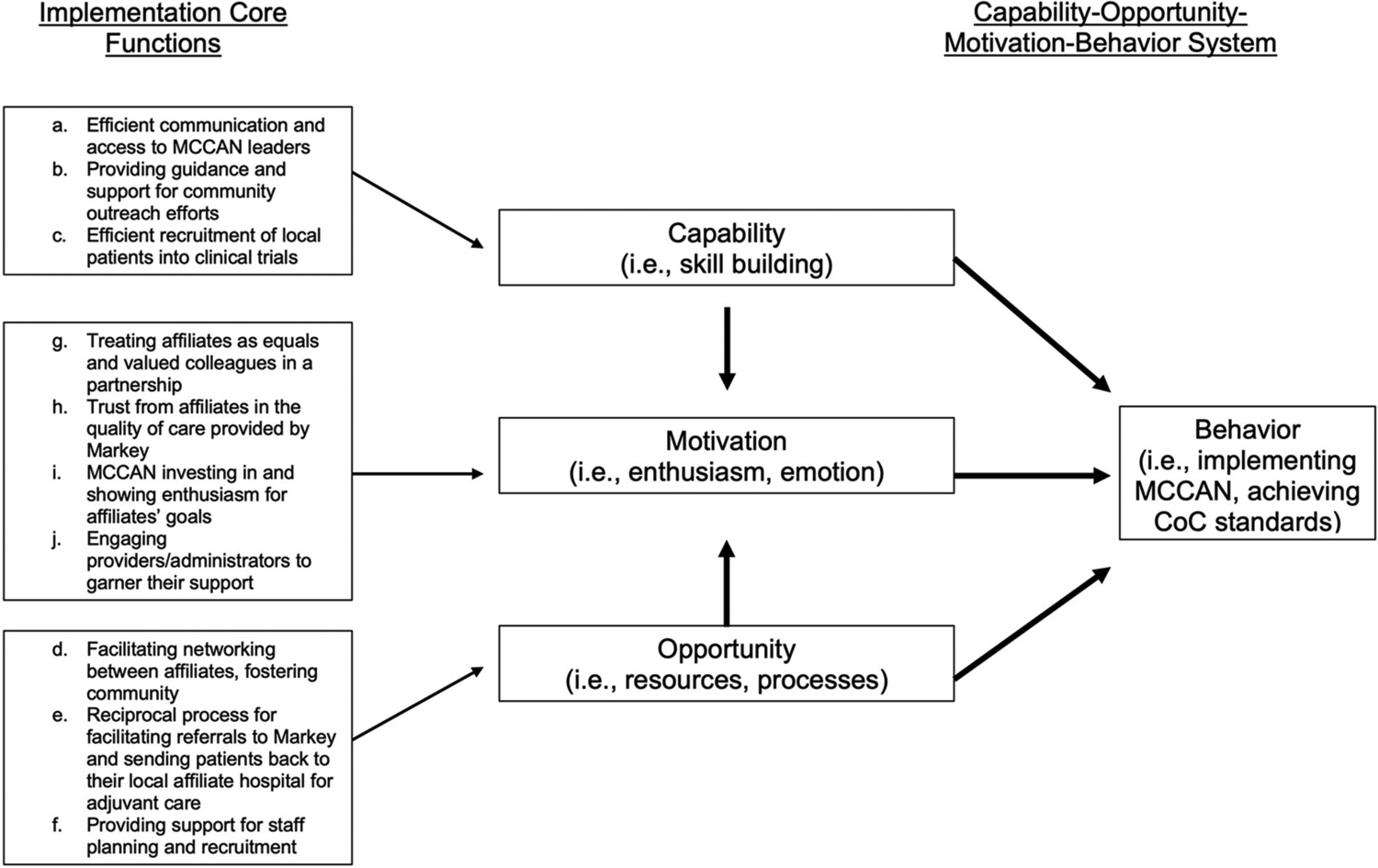
Implementations core functions mapped onto capability-opportunity- motivation-behavior system theory of change [19].

We identified 10 implementation core functions as necessary and collectively sufficient to achieve CoC standards and describe them in detail below (Table 3; designated below as a-j). We propose that these core functions represent MCCAN giving affiliates the capability, opportunity, and motivation required to achieve CoC standards. The capability-opportunity-motivation-behavior (COM-B) system is based on a synthesis of 33 psychological theories that collectively suggest that behavior (in this case, MCCAN implementation) becomes possible only when the capability, opportunity, and motivation exist to do so (Figure 2; i.e., theory of change) (17).

### Capability – Implementation Functions a-c

MCCAN increased the capability of affiliates to achieve accreditation standards by a) *efficient communication and access to MCCAN leaders*, b) *providing guidance and support for community outreach efforts*, and c) *efficient recruitment of local patients into clinical trials*.

a) *Efficient communication and access to MCCAN leaders facilitating access to information and resources*. MCCAN’s wealth of expertise made them the appropriate “first phone call” (P1.1) for affiliates when they encountered problems or had questions. MCCAN leadership created a culture of open communication so that affiliates were comfortable coming straight to them and “not being afraid to pick up the phone and call them” (P4.4) when needed to access information or obtain resources.

b) *Providing guidance and support for community outreach*, and c) *efficient recruitment of local patients into clinical trials*. The CoC had specific standards related to community outreach and clinical trial enrollment which were referred to as challenging standards to achieve. MCCAN made it more feasible for rural programs to achieve these standards by providing resources (i.e. financial: “money… they match toward prevention, screening” (P4.1)) and information (i.e. lists of available clinical trials (P3.2)) to programs so that they may overcome barriers to achieving these standards.

### Opportunity – Implementation Functions d-f

MCCAN increased the opportunity for affiliates to achieve accreditation standards by d) *facilitating networking between affiliates*, e) *reciprocal process for facilitating referrals to Markey and sending patients back to their local affiliate hospital for adjuvant care*, and f) *providing support for staff planning and recruitment*.

d) *Facilitating networking between affiliates, fostering community within the Network*. It is often valuable for small hospitals to seek guidance from similar sized hospitals which are more likely to have experienced similar challenges. MCCAN served as the hub that “spokes [the] network together with best practices on what’s working and what’s not” (P1.1) through formal and informal networking activities to facilitate connections “so that we can talk about things that I feel are a little more unique to rural facilities [at the MCCAN annual meeting]” (P5.1).

e) *Reciprocal process for facilitating referrals to Markey and sending patients back to their local affiliate hospital for adjuvant care*. MCCAN intended to provide services to patients that are not accessible in their communities with the ultimate goal of allowing patients to remain in their communities for the majority of their care. Concerns that MCCAN may “steal [affiliates’] patients” (P2.1–3.1, P4.4), made the commitment between parties to “get [patients] through the part that they need to be through” away from home, and to “refer them back” (P4.1) to their communities was critical for increasing the acceptability of MCCAN affiliation among stakeholders.

f) *Providing support for staff planning and recruitment*, especially early in the accreditation process. The accreditation process often requires hiring new positions to meet standards, especially the CTR role, adding to the barriers for rural hospitals, often under-staffed to begin with, to achieve accreditation. MCCAN provided networking (i.e., “Behind the Scenes” meetings where community hospital staff could meet their counterparts at Markey and discuss their roles) and recruitment support (i.e., providing job descriptions and job responsibility documents to fill new roles).

### Motivation – Implementation Functions g-j

MCCAN increased the motivation of affiliates to achieve accreditation standards by g) *treating affiliates as equals in a partnership*, h) *developing trust from affiliates in the care quality of Markey*, i) *investing in affiliates’ goals*, and j) *engaging affiliate stakeholders to garner their support for implementation of CoC standards*.

g) *Treating affiliates as equals and valued colleagues in a partnership*, and h) *developing trust from affiliates in the quality of care provided by Markey*. MCCAN created a culture in which input was valued from all members and the relationship is defined by mutual respect and reciprocity. As one affiliate described, “establish[ing] that relationship” was critical, and “MCCAN is UK. So they’re like top notch… but they are just great with that one on one… that connection, that’s the best part of [affiliation]” (P4.4). However, it was essential for stakeholders at affiliate hospitals to perceive affiliation as valuable to their program and patients. Pursuing accreditation was only acceptable if there was trust that mentors at MCCAN can provide exceptional care and offer affiliates sound advice and resources to improve the quality of care at their own institutions.

i) *MCCAN investing in and showing enthusiasm for affiliates’ goals* and j) *engaging providers and administrators in the accreditation process to garner their support*. The accreditation process was a significant commitment taking “about a year and a half,” so willingness of key stakeholders such as clinicians and administrators to participate is crucial (P3.2). MCCAN was successful in engaging stakeholders because of the enthusiasm and motivation from its leaders. As one affiliate describes, the MCCAN medical director was “very excited about all this… you need that person that motivates everybody” (P1.2). MCCAN elicited both institutional and individual goals and needs to tailor support and resources to achieve these goals and accreditation. According to one affiliate, “the things we picked … as goals… were always our own… we were able to do the things that were important to us” (P4.1). Goals that were tailored to affiliate hospitals created a unique relationship with MCCAN’s support and resources.

## DISCUSSION

We identified MCCAN’s core functions—the features of MCCAN that were collectively sufficient to support affiliate hospitals in achieving CoC accreditation and, in turn, improve care quality for rural patients. Specifically, we found that affiliate hospitals improved quality when they balanced the benefits of affiliation with MCCAN (i.e., providing better patient care) against the dependence that comes with affiliation (e.g., relying on MCCAN for the expertise required to achieve CoC accreditation), and that MCCAN gave affiliates the capability (e.g., support for community outreach), opportunity (e.g., reciprocal patient referral processes), and motivation (e.g., investing in affiliates’ goals) required to achieve CoC standards. Although CoC accreditation is not synonymous with high-quality care, our previous work suggests that CoC-accredited hospitals provide higher-quality care than non-CoC accredited hospitals (6). Accreditation requires that hospitals provide specific clinical and supportive services, and pursuit of accreditation demonstrates a program’s commitment to high-quality care delivery (18). Research has shown that cancer patients in rural areas face limited access to medical and oncology providers, long travel times, and low recruitment to clinical trials, all of which affect quality of care and health outcomes (19). However, a recent article reported CoC accreditation as an independent predictor of performance on four evidence-based quality measures in multivariable models controlling for patient rurality, hospital rurality, hospital bed size and hospital accreditation status (20). This suggests that improving the quality of care at rural hospitals through assisting in the CoC accreditation process may result in better outcomes for rural cancer patients. Indeed, Unger et al. found that participation in clinicals trial reduced disparities in cancer outcomes between rural and urban patients, providing additional evidence that improving access to uniform, high-quality treatment in rural hospitals may be an effective strategy (21). Performance on evidence-based quality measures, as well as expectations regarding patient care protocols and operative standards are all part of the CoC accreditation standards and therefore provide a mechanism to support more uniform, high-quality treatment (18). Thus, we suggest that the identified intervention core functions have made MCCAN effective at improving care quality in their state, and that maintained in adaptation, they can improve care quality in other contexts.

While the intervention core functions underscore MCCAN’s ability to improve care quality, the implementation core functions outline the collectively sufficient activities necessary of MCCAN to help affiliate hospitals actually achieve CoC standards. A few of these core functions relate to specific standards (implementation core functions b-c), indicating that some standards may be especially difficult for rural facilities to meet. For instance, the CoC requires accredited programs to host annual community outreach cancer prevention and screening events. The CoC also requires a percentage of patients to be enrolled in cancer-related research studies (18). Participants cited MCCAN’s assistance with these two standards in the form of financial and informational support for community outreach and clinical trial accrual as critical to their ability to achieve accreditation. At present, the CoC requires all accredited programs to be compliant with these standards. Our results suggest that previously unaccredited facilities struggle disproportionately with these specific standards compared to others, and without support from MCCAN, accreditation is not achievable. This may partially explain why currently, only 16% of non-metro/rural hospitals are CoC-accredited [compared to 82% of metro hospitals (22)], and provides evidence that interventions like MCCAN are needed around the country to increase feasibility of achieving accreditation for rural hospitals. Most of the implementation strategies are focused on building and maintaining relationships between MCCAN and affiliates (Figure 2). Pursuing CoC accreditation is a major undertaking for any facility, and rural facilities face additional barriers. It is essential that any intervention attempting to decrease these barriers does so with partnership in mind.

This study produced evidence regarding MCCAN’s intervention and implementation core functions. The identified core functions are complete, comprehensive, and achievable for those who seek to adapt MCCAN without compromising its effectiveness. However, it is important to note that some of the core functions may be particularly challenging to operationalize in new contexts. For example, we identified trust from affiliates in the quality of care provided at MCCAN’s hub comprehensive cancer center to which affiliates could refer patients as an implementation core function (Table 3e). Developing trust between community hospitals and a hub comprehensive cancer center in contexts where trust was previously lacking is likely to be very challenging. Additionally, significant resources are required of potential adapters to successfully achieve the core functions (i.e., educational materials, clinical expertise, specialized services, etc.), potentially compromising core functions in particularly resource-poor settings. Despite these potential challenges, the successful adaptation of MCCAN could change the paradigm of how rural hospitals deliver cancer care to their patients, shifting the focus from the traditional centralization of cancer care at high-volume facilities recommended by many in the literature (23–32) to a system in which rural hospitals are empowered and enabled to provide the highest-quality care at their own facilities for many types of common cancers, decreasing barriers and allowing rural patients to access high-quality care close to home.

This work contributes to a growing body of literature that identifies core functions of evidence-based interventions (11–13, 33, 34). The process of identifying core functions of an evidence-based intervention *post-hoc* has been previously described (12), though this is among the first application of these methods to prospectively inform adaptations to an existing intervention for use in alternative contexts. These methods can be used to adapt other interventions to address rural cancer disparities and as a prototype for replicating MCCAN.

Our study makes several policy, research, and conceptual contributions. In terms of policy, our findings suggest that the core functions should guide the process of scaling up the MCCAN model across the US. Our study also advances state-of-the-science methods of identifying core functions developed by SAB and her colleagues. In applying these innovative methods, we have further codified and refined the process. Specifically, this study sheds light on the need to identify core functions related to an intervention’s implementation as well as the core functions of the intervention itself. To conceptualize implementation core functions, implementation theories and theoretical frameworks and models such as COM-B (17) may be particularly useful as theories of change. Conceptually, our study suggests that, for interventions that facilitate coordination among multiple institutions, as is the case with MCCAN, organization theories are highly relevant given their focus on conditions in the outer setting that can be harnessed to improve care (35). We also demonstrated the utility of organization theory in serving as theories of change for organization-level interventions such as MCCAN.

### Limitations

This study has several important limitations. We did not gather information from affiliate hospitals who had achieved CoC-accreditation prior to affiliation with MCCAN nor did we gather information from rural hospitals who were unable to achieve CoC-accreditation despite committing to the process. Without these perspectives, we are not able to fully understand the benefits MCCAN provides to hospitals that are already accredited and what incentivizes their decision to join the network. Additionally, we lack understanding of the barriers to achieving accreditation that were too great for some hospitals to overcome, even with the support of MCCAN. Future research should explore whether and how hospitals who do not achieve accreditation differ from the affiliates that secured CoC-accreditation while relying on the scaffolding provided by MCCAN.

Another limitation of this study is the small number of participants included in the analysis, though this was by design to meet our study objectives. We were purposeful about selecting representatives from the five hospitals that were not CoC accredited at the time of joining MCCAN because they mostly closely matched the rural hospitals that would be targets of the adapted intervention. It is possible that the eight representatives from the five targeted affiliate hospitals who chose to participate had a perception of MCCAN and its core functions which differed from those three representatives who chose not to participate, but it is also likely these representatives were the most involved in MCCAN implementation and therefore were able to contribute the in depth information we were seeking. Futhermore, the themes which gave rise to the core functions were common amongst multiple interviews in our sample, and we confirmed validity of the results through multiple mechanisms (i.e., confirming face validity with MCCAN leadership and mapping core functions onto existing organization theory of change). Thus, we are confident that we have successfully identified the core functions of the MCCAN intervention.

### Future Directions

If MCCAN is to be successfully adapted, the next step is to identify the contextual differences between Kentucky and the new contexts for the adaptation. Contextual differences can be addressed with adaptations to MCCAN’s forms while preserving the core functions identified in this analysis.

The state of Iowa is an ideal setting in which to pilot the scale-up of the MCCAN model. Like Kentucky, Iowa is a rural state with a high cancer incidence and almost no CoC accredited hospitals in non-metro/rural areas for its 40% rural population (36, 37). Both states have a single National Cancer Institute (NCI)-designated Cancer Center and similar number of hospitals (37, 38). However, MCCAN’s scalability to a rural cancer hospital network in Iowa is limited by systematic differences between Kentucky and Iowa. For example, Markey Cancer Center (Kentucky’s NCI-designated Cancer Center) is centrally located and a major referral center across Kentucky, whereas Iowa’s NCI-designated Cancer Center is located near the state’s eastern border, thus hospitals across the state may choose to refer patients to other large cancer centers in neighboring states that are geographically closer rather than to Iowa’s NCI-designated Cancer Center. Without adaptation to Iowa’s unique context, MCCAN may not be viewed as appropriate, acceptable, or feasible in Iowa and therefore be poorly implemented. Further, unadapted, MCCAN may lack the features required to improve cancer care quality in Iowa. Thus, the next steps of our project will follow Kirk et al.’s method (12) of adaptation – i.e., addressing systematic differences between Kentucky and Iowa with adaptations identified through rigorous qualitative methods while preserving MCCAN’s originally demonstrated levels of effectiveness. In addition, we will evaluate the costs and benefits to hospitals in pursuing and achieving the CoC standards.

## CONCLUSION

Much research has focused on the potential benefits of centralizing cancer care to high-volume, urban cancer centers, yet this strategy largely ignores the challenges faced by rural populations and the desires of many cancer patients to receive care closer to home. The MCCAN model presents a more equitable strategy of extending the resources and expertise of large cancer centers to assist smaller community hospitals in achieving the evidence-based standards for cancer care. Using rigorous qualitative methods, we found that rural cancer care disparities can be addressed by aiding rural hospitals in balancing the benefits of affiliation with a quality-focused network against the dependence that it requires, and giving rural hospitals the capability, opportunity, and motivation to achieve CoC quality standards. Distilling this complex, multi-level intervention into these core functions has positioned us (and others) to adapt the MCCAN model to address cancer disparities in other rural contexts.

## Supplementary Material

Supplementary Material

## Figures and Tables

**TABLE 1 |:** Participant roles (*n* = 8).

Participant ID (institution ID.individual ID)	Hospital role
1.1	Oncology service line director
1.2	Medical oncologist, Cancer liaison physician (CLP), CoC chair
2.1–3.1	Cancer program coordinator
3.2	Medical oncologist, CLP
4.1	Cancer program coordinator
4.3	Family medicine physician, CLP
4.4	Certified tumor registrar
5.1	Oncology service line director

**TABLE 2 |:** Representative participant quotes for intervention core functions.

a. Providing expertise about CoC standards	P4.1: “Or, um, you know, if we had a meeting and we need to steer in a different direction, having that guidance really did help from people that knew how, um, the commission on cancer worked and what they were really looking for” P4.3: “…they, they do supply… people who know what, what they’re doing… especially some of the things that we wouldn’t think about.”
b. Providing a plan and framework for becoming accredited	P4.3: “so the parts that I have, uh, you know, that seem essential, uh, to help you bring up to speed… Somebody that knows how to give you the stepping stones, uh, to perform all that stuff [accreditation]” P4.4: “even if it’s something they’ve never done before… but I have no doubt I could call them and be like, this has come up. I don’t know what to do. How do we need to handle this?”
c. Establishing a culture of data-driven quality improvement	P1.2: “you get to demonstrate… here’s one need we have, but also here’s what we’re working with… The two biggest things is how are your patients doing? Are you doing like the standard of care?… And it helps you kind of measure… is there something that the hospital needs to take a step toward? Well, let’s bring in somebody that can help us and then take it from there”
d. Prioritizing the role of the CTR in using data to drive program enhancements	P1.1: “The CTR role, um, I feel like that is a vital role… CTRs are just very valuable.” P5.1: “…when I started this process… I didn’t even know what a CTR was……we don’t have a CTR were likely not going to be able to recruit a CTR… And so, um, at that time, when they [MCCAN] hired some folks… that could help some of the small rural facilities fill and meet those roles… if we hadn’t have had that piece of it, um, I’m not, I’m really not sure what we would have done at the time”
e. Establishing a shared goal of providing the best care for patients	P1.2: “you realize that everybody’s trying to work toward the same goal. And like, that’s, I mean, that’s what makes it run. It’s everybody… that you can feel comfortable… sending your patients to” P4.4: “I kind of feel like MCCAN, you know, that’s what we are doing is providing the best care possible when you look at it as a whole and not just the disease [cancer] itself”
f. Educating providers to help them provide better care and help patients make informed decisions	P2.1–3.1: “as far as all the [educational] activities, I think just because they they’re specific [why they are beneficial], it’s not a broad range of subjects that they’re going to talk about during a 1 hour it’s it’s, they’ve pinpointed it down to a specific subject.” P4.4: “I’ve seen patients say that, you know, I chose the lumpectomy because it was the least invasive, but he didn’t tell me if I chose that I would have to have radiation…. I mean, I’m not saying that’s bad on physician’s part that they just wasn’t educated, I suppose… that’s [MCCAN’s educational offerings] just, that’s, that’s just helped so much… they’re [the patients] getting better care because they know more.”
g. Helping patients feel secure in their choice to seek care locally	P1.2: “…if you were affiliated with this larger center, you know, that speaks to the care you’re providing and that’s goes along with the commission on cancer accreditation…I think that helps for patients because I’ve had some people come and see me just specifically, because I was a Markey affiliate” P4.4: “Everybody knows the UK is here. You know, that makes them feel safer, I suppose… so by becoming affiliated with MCCAN… it states that on there, you know, not only the COC accreditation… they know that you’re going to have to give your patients the highest level of care”
h. Allowing patients to access programs and specialized services not locally available	P4.1: “we’ve been able to connect patients with genetic counseling and other services that we just would not have had prior” P1.2: “And you know… we’re still gonna need the different radiation procedures that a university hospital provides that we’re just not going to have in the community… if you can be in a network or you can have access to those services and, you know, have an easier way of getting those services facilitated, like why not?” P5.1: some financial impact things that way as a small community hospital, that we, we didn’t have deep enough pockets to provide…we were able to work with the, the MCCAN group to fill that void”

**TABLE 3 |:** Representative participant quotes for implementation core functions.

a. Efficient communication and access to MCCAN leaders facilitating access to information and resources	P1.1: “I have always felt that they were very supportive and…if I felt like I was in a roadblock with something…usually if I feel like I’ve exhausted all my resources they’re [MCCAN] my first phone call” P4.4: “You know, having that communication, that open communication and not being afraid to pick up the phone and call them… they are just great with that one on one, you can, I mean, you text them, you call them, I guess that open door. That, that connection, that’s the best part of it”
b. Providing guidance and support for community outreach efforts	P1.1: “that’s one another great thing about the affiliate network is the support that they will provide with screening initiatives…to get out in the community to target the uninsured or the under-insured” P4.1: “MCCAN has helped us put together programs, um, that have made us be more engaged in the community to get more people, to be interested in screenings and prevention”
c. Efficient recruitment of local patients into clinical trials	P3.2: “UK MCCAN have a list of longer clinical trials where we can enroll our patients in, or they can even help us to suggest clinical trial outside of UK and MCCAN, if this is not available” P4.4: “Um, clinical trials, that’s one of the things we have the hardest time with…but like MCCAN offers you things like, uh, you can refer your patients to them for a clinical trial, even if it’s a teleconference”
d. Facilitating networking between affiliates, fostering community	P1.1: “I don’t mind to share, you know…it’s really nice to get the support from the other hospitals because, you know, we don’t feel like we’re in competition with each other… We want to see everyone succeed” P5.1: “…it’s nice to have that opportunity to come together in smaller groups with folks that are more closely related to your, your size…Markey gave me your name…Can you tell me what you did about this…?”
e. Reciprocal process for facilitating referrals to Markey and sending patients back to local hospital for adjuvant care	P1.2: “And I think…the affiliate network helps the larger hospital by getting patients that need care at a larger hospital. But…then the people come back to the community. And I think that’s the overall goal for most people” P2.1–3.1: “they [MCCAN leadership] would go to that person [provider] say, Hey, you know, we have this affiliation agreement. You need to make sure that patient gets sent back to that facility for their treatments” P4.1: “…they [MCCAN] are not here to steal our patient. They’re here to take them, get them through the part that they need to be through, and then they’re going to refer them back”
f. Providing support for staff planning and recruitment	P5.1: “the biggest resource and value was staffing. Being able to…kind of get up off the ground and get running and get started and helping us grow our, our program…we might not be here if it really wasn’t for that”
g. Treating affiliates as equals in a partnership	P4.4: “I go in there, all these suits and ties and I’d kind of be intimidated, but you know…They’re just smart in what they do, but I’m smart in what I do. They can’t do what I do and I can’t do what they do. And they don’t treat you like that”
h. Trust from affiliates in the quality of care provided by Markey	P3.2: “what drive the patient to go to UK MCCAN is a quality of care. So the quality of care, you know, …if the quality of care drops, then…you know, wouldn’t lead to a successful, uh, merge or affiliation here”
i. MCCAN leadership investing in and showing enthusiasm for affiliates’ goals	1.2: “Dr. Mullet. Like…this is his jam doing this whole affiliate stuff…so he is very excited about all this… It helps when he’s so into it…you need that person that motivates everybody” 4.1: “you know, the things that we picked as far as goals…were always our own…we were able to do the things that were important to us …” P4.4: “you know, they’re [MCCAN] excited about the program and they want to put all those things in place and they’ll work close with them to, to make sure, you know, we’re doing that”
j. Engaging providers/administrators to garner their support for intervention	P3.2: “physicians should be willing to participate…took around year and a half to get CoC accreditation…So administration willingness to participate also…trying to convince them [physicians and administrators]… I think that’s the biggest challenge here” P4.1: “they’ve helped bring in some other members that you wouldn’t typically see…their cancer liaison physician currently, he’s a family practice doctor…it’s brought other people from other departments in …it’s helped us offer so much more for our patients”

## Data Availability

The raw data supporting the conclusions of this article will be made available by the authors, without undue reservation.

## References

[1] Charlton M, Schlichting J, Chioreso C, Ward M, Vikas P. Challenges of rural cancer care in the United States. Oncology. (2015) 29:633–40. 26384798

[2] Diaz A, Pawlik TM. Rural surgery and status of the rural workplace: hospital survival and economics. Surg Clin North Am. (2020) 100:835–47. doi: 10.1016/j.suc.2020.05.009 32882166

[3] Aboagye JK, Kaiser HE, Hayanga AJ. Rural-urban differences in access to specialist providers of colorectal cancer care in the united states: a physician workforce issue. JAMA Surg. (2014) 149:537–43. doi: 10.1001/jamasurg.2013.5062 24740165

[4] Weigel PAM, Ullrich F, Ward MM. Rural bypass of critical access hospitals in Iowa: do visiting surgical specialists make a difference? J Rural Health. (2018) 34(Suppl 1):s21–s9. doi: 10.1111/jrh.12220 27677870

[5] Shulman LN, Browner AE, Palis BE, Mallin K, Kakade S, Carp N, Compliance with cancer quality measures over time and their association with survival outcomes: the commission on cancer’s experience with the quality measure requiring at least 12 regional lymph nodes to be removed and analyzed with colon cancer resections. Ann Surg Oncol. (2019) 26:1613–21. doi: 10.1245/s10434-019-07323-w 30927195

[6] Charlton M, Kahl A, Gao X, Schroeder M, Kapadia M, Lizarraga I. Commentary and complementary data to add to “compliance with cancer quality measures over time and their association with survival outcomes: the commission on cancer’s experience with the quality measure requiring at least 12 regional lymph nodes to be removed and analyzed with colon cancer resections. Ann Surg Oncol. (2020) 27:1306–7. doi: 10.1245/s10434-019-08150-9 31898095 PMC7112949

[7] Tucker TC, Charlton ME, Schroeder MC, Jacob J, Tolle CL, Evers BM, Improving the quality of cancer care in community hospitals. Ann Surg Oncol. (2021) 28:632–8. doi: 10.1245/s10434-020-08867-y 32712893 PMC7854809

[8] Commision on Cancer. Commission on Cancer 2022. Available online at: https://www.facs.org/quality-programs/cancer/coc.

[9] Cohen DJ, Crabtree BF, Etz RS, Balasubramanian BA, Donahue KE, Leviton LC, Fidelity versus flexibility: translating evidence-based research into practice. Am J Prev Med. 2008 35(5 Suppl):S381–9. doi: 10.1016/j.amepre.2008.08.005 18929985

[10] Kirk MA, Moore JE, Wiltsey Stirman S, Birken SA. Towards a comprehensive model for understanding adaptations’ impact: the model for adaptation design and impact (MADI). Implement Sci. (2020) 15:56. doi: 10.1186/s13012-020-01021-y 32690104 PMC7370455

[11] Perez Jolles M, Lengnick-Hall R, Mittman BS. Core functions and forms of complex health interventions: a patient-centered medical home illustration. J Gen Intern Med. (2019) 34:1032–8. doi: 10.1007/s11606-018-4818-7 30623387 PMC6544719

[12] Kirk MA, Haines ER, Rokoske FS, Powell BJ, Weinberger M, Hanson LC, A case study of a theory-based method for identifying and reporting core functions and forms of evidence-based interventions. Transl Behav Med. (2021) 11:21–33. doi: 10.1093/tbm/ibz178 31793635 PMC7877297

[13] Kirk MA, Hanson LC, Weinberger M, Haines ER, Rokoske FS, Powell BJ, Pilot test of an adapted intervention to improve timeliness of referrals to hospice and palliative care for eligible home health patients. J Palliat Med. (2019) 22:1266–70. doi: 10.1089/jpm.2018.0504 31090487 PMC7364317

[14] Gao X, Schroeder MC, Lizarraga IM, Tolle CL, Mullett TW, Charlton ME. Improving cancer care locally: study of a hospital affiliate network model. J Rural Health. (2021). doi: 10.1111/jrh.12639. [Epub ahead of print]. PMC9189248 34897807

[15] Proctor E, Silmere H, Raghavan R, Hovmand P, Aarons G, Bunger A, Outcomes for implementation research: conceptual distinctions, measurement challenges, and research agenda. Adm Policy Ment Health. (2011) 38:65–76. doi: 10.1007/s10488-010-0319-7 20957426 PMC3068522

[16] Pfeffer J, Salancik GR. The External Control of Organizations: A Resource Dependence Perspective. Stanford: University Press (2003).

[17] Michie S, van Stralen MM, West R. The behaviour change wheel: a new method for characterising and designing behaviour change interventions. Implement Sci. (2011) 6:42. doi: 10.1186/1748-5908-6-42 21513547 PMC3096582

[18] Commission on Cancer. Optimal Resources for Cancer Care (2020 Standards). Chicago, IL: American College of Surgeons (2021).

[19] Levit LA, Byatt L, Lyss AP, Paskett ED, Levit K, Kirkwood K, Closing the rural cancer care gap: three institutional approaches. JCO Oncol Pract. (2020) 16:422–30. doi: 10.1200/OP.20.00174 32574128

[20] Schroeder MC, Gao X, Lizarraga I, Kahl AR, Charlton ME. The impact of commission on cancer accreditation status, hospital rurality, and hospital size on quality measure performance rates. Ann Surg Oncol. (2022) 29: 2527–36. doi: 10.1245/s10434-021-11304-3 35067792 PMC11559211

[21] Unger JM, Moseley A, Symington B, Chavez-MacGregor M, Ramsey SD, Hershman DL. Geographic distribution and survival outcomes for rural patients with cancer treated in clinical trials. JAMA Netw Open. (2018) 1:e181235. doi: 10.1001/jamanetworkopen.2018.1235 30646114 PMC6324281

[22] Bilimoria KY, Bentrem DJ, Stewart AK, Winchester DP, Ko CY. Comparison of commission on cancer-approved and -nonapproved hospitals in the United States: implications for studies that use the national cancer data base. J Clin Oncol: Off J Am Soc Clin Oncol. (2009) 27:4177–81. doi: 10.1200/JCO.2008.21.7018 19636004

[23] Archampong D, Borowski D, Wille-Jørgensen P, Iversen LH. Workload and surgeon’s specialty for outcome after colorectal cancer surgery. Cochrane Database Syst Rev. (2012) 14:CD005391. doi: 10.1002/14651858.CD005391.pub3 PMC12076000 22419309

[24] Billingsley KG, Morris AM, Green P, Dominitz JA, Matthews B, Dobie SA, Does surgeon case volume influence nonfatal adverse outcomes after rectal cancer resection? J Am Coll Surg. (2008) 206:1167–77. doi: 10.1016/j.jamcollsurg.2007.12.042 18501815 PMC3103396

[25] Hodgson DC, Zhang W, Zaslavsky AM, Fuchs CS, Wright WE, Ayanian JZ. Relation of hospital volume to colostomy rates and survival for patients with rectal cancer. J Natl Cancer Inst. (2003) 95:708–16. doi: 10.1093/jnci/95.10.708 12759388

[26] Huscher CG, Bretagnol F, Corcione F. Laparoscopic colorectal cancer resection in high-volume surgical centers: long-term outcomes from the LAPCOLON group trial. World J Surg. (2015) 39:2045–51. doi: 10.1007/s00268-015-3050-4 25820910

[27] McGrath DR, Leong DC, Gibberd R, Armstrong B, Spigelman AD. Surgeon and hospital volume and the management of colorectal cancer patients in Australia. ANZ J Surg. (2005) 75:901–10. doi: 10.1111/j.1445-2197.2005.03543.x 16176237

[28] Meyerhardt JA, Tepper JE, Niedzwiecki D, Hollis DR, Schrag D, Ayanian JZ, Impact of hospital procedure volume on surgical operation and long-term outcomes in high-risk curatively resected rectal cancer: findings from the Intergroup 0114 Study. J Clin Oncol: Off J Am Soc Clin Oncol. (2004) 22:166–74. doi: 10.1200/JCO.2004.04.172 14701779

[29] Ptok H, Marusch F, Kuhn R, Gastinger I, Lippert H. Influence of hospital volume on the frequency of abdominoperineal resection and long-term oncological outcomes in low rectal cancer. Eur J Surg Oncol. (2007) 33:854–61. doi: 10.1016/j.ejso.2006.12.020 17933024

[30] Sahni NR, Dalton M, Cutler DM, Birkmeyer JD, Chandra A. Surgeon specialization and operative mortality in United States: retrospective analysis. Bmj. (2016) 354:i3571. doi: 10.1136/bmj.i3571 27444190 PMC4957587

[31] Finks JF, Osborne NH, Birkmeyer JD. Trends in hospital volume and operative mortality for high-risk surgery. N Engl J Med. (2011) 364:2128–37. doi: 10.1056/NEJMsa1010705 21631325 PMC3150488

[32] Sheetz KH, Dimick JB, Nathan H. Centralization of high-risk cancer surgery within existing hospital systems. J Clin Oncol: Off J Am Soc of Clin Oncol. (2019) 37:3234–42. doi: 10.1200/JCO.18.02035 PMC7351344 31251691

[33] Anderson DM, Birken SA, Bartram JK, Freeman MC. Towards more systematic adaptation of water, sanitation, and hygiene interventions: an adaptation model and scoping review of key concepts and tools. (2022). 10.3389/frhs.2022.896234 PMC10012759 36925880

[34] Griesemer I, Birken SA, Rini C, Maman S, John R, Tatcher K, Mechanisms to enhance racial equity in healthcare: developing a model to facilitate translation of an evidence-based intervention. (2022). 10.1016/j.ssmqr.2022.100204 PMC10361418 37483653

[35] Birken SA, Bunger AC, Powell BJ, Turner K, Clary AS, Klaman SL, Organizational theory for dissemination and implementation research. Implement Sci. (2017) 12:592. doi: 10.1186/s13012-017-0592-x PMC5427584 28499408

[36] U.S. Cancer Statistics Working Group. U.S. Cancer Statistics Data Visualizations Tool, based on 2020 submission data (1999–2018): U.S. Department of health and human services, centers for disease control and prevention and national cancer institute. Available online at: www.cdc.gov/cancer/dataviz, released in June 2021.

[37] U.S. Department of Health and Human Services. Rural Health Information Hub. State Guides: Iowa. [updated February 2021. Available online at: https://www.ruralhealthinfo.org/states/iowa

[38] U.S. Department of Health and Human Services. Rural Health Information Hub. State Guides: Kentucky. updated March 2021. Available online at: https://www.ruralhealthinfo.org/states/kentucky

