# Supplementary material for: Identifying Core Functions of an Evidence-Based Intervention to Improve Cancer Care Quality in Rural Hospitals
Source: Front Health Serv. Author manuscript; Available in PMC 2022 Sep 30. (PMC9524475; doi:10.3389/frhs.2022.891574)
Supplement: Supplementary file 1 [file NIHMS1838589-supplement-Supplementary_Material.pdf]

Supplementary Material

Supplemental Table 1. Document describing MCCAN's forms

Markey Cancer Center Affiliate Network (MCCAN)	
**WHO/WHERE: People/places essential to implementation**	**ROLES: Purpose and function of each place or role involved in the intervention**
**MCCAN - Where the intervention was developed and supported**	
Medical Director	Provides clinical leadership and supports physician champions at each site. Offers one-on-one mentoring for new CLPs and provides updates on new standards and benchmarks. Serves as a liaison for clinical outreach and represents MCCAN interests to faculty and administration. Works with UK leadership to maintain visibility of program internally and externally.
Administrative Director	Oversees MCCAN operations and provides senior-level support. Manages contracts, budgets, and personnel. Primary administrative contact for sites. Works with marketing and public relations for brand standards.
Quality Director	Represents MCCAN on quality performance issues. Supervises quality and affiliate liaison teams. Serves as liaison between CoC and MCCAN. Identifies resources and contacts at Markey to meet site needs. Represents MCCAN for Markey cancer committee and quality meetings. Assists with development of nursing education. Attends CoC surveys and oversees resolution of program deficiencies.
Quality Assurance Coordinator	Develops timeline for sites to achieve/maintain accreditation, assists site in developing/completing Quality Improvement studies, provides services tailored to goals and challenges of each site, facilitates communication among sites to support the development and implementation of new initiatives, provides updates of sites at monthly MCCAN team meeting.
Certified Tumor Registrar Compliance Coordinator	Provides training, coaching, and mentoring on NCDB tools, quality measures and updates of standards for tumor registrars; conducts review of sample pathology reports; develops algorithms for clinical measures using abstract data to provide sites progress reports.
Affiliate Liaison	Facilitates incoming referrals to Markey and obtains necessary patient records. Conducts pre-appointment call with referred patients, works with patient and affiliate site to arrange for treatment close to home when appropriate. Responds to complaints from MCCAN sites or patients.
Education Director	Creates, facilitates, and develops educational programs for affiliate sites. Conducts needs assessments, produces technical aspects of events, maintains and updates MCCAN web portal with educational and community resources available to sites. Supervises education team.
**Community Hospitals Across Kentucky - Where the intervention was implemented**	
Clinical Personnel (physicians)	Develops and establishes cancer committee, tumor boards, care delivery consistent with clinical guidelines and performance measures.

Administrative personnel (chief executive officers, managers, staff)	Oversees quality improvement activities, data collection and monitoring, documentation of cancer committee and tumor boards, and all other accreditation activities.	
**WHAT: the "ingredients" essential to the success of the intervention**		
Providing quality improvement support and educational/ training resources	Providing access to specialized clinical expertise<br><br>Providing expertise in accreditation standards	Advocacy and expertise to assist hospital administrators in making requests for needed resources, staff/positions, and equipment
Offering access to clinical services not available locally	Gap analyses/needs assessments	Educational sessions to meet the needs of clinicians at affiliate sites
Ongoing support beyond accreditation	Use of UK logo and co-sponsored community events	Round table with sites to share strategies and best practices
**WHY: the reason or purpose for the intervention**		
To improve quality<br><br>To improve patient satisfaction and financial performance	To assist hospitals in reaching and maintaining CoC accreditation standards	To improve staff morale<br><br>To improve the referral process to the UK Markey Cancer Center
**HOW: methods and conditions necessary to implement the intervention**		
Building Trust	Strong interpersonal relationships between MCCAN and the hospitals	Sending patients back to MCCAN sites for local treatment and follow-up care
Providing Tailored Support, Structure, and Services	Goals, strategies, and training plans based on individualized assessments<br><br>Filling the gaps by providing access to resources within the network<br><br>Champions for success<br><br>Patient support	Individualized support to make necessary changes to meet accreditation requirements<br><br>Clear, consistent communication<br><br>Liaison to the CoC<br><br>Long-term commitment<br><br>Brand support
Changing Culture, not just Strategies	Innovative support to reach new goals<br><br>Strategy sharing among affiliates	Validity, quality, and trust between patients and staff<br><br>Continuous quality improvement

Supplemental Table 2. Excerpt of MCCAN core functions interview guide relevant to identifying the core functions of the intervention. Interview guide developed based on the methods described by Kirk et al. (12)

Part I. Identification of MCCAN Core Functions and Forms

The first phase in our research is to learn more about MCCAN. Specifically, I want to: 1) understand the current state of cancer care at your hospital, 2) understand how MCCAN activities and services assist in achieving and maintaining CoC accreditation, 3) identify essential MCCAN activities. I wanted to speak to you to hear your perspective on these issues. I'm then going to use what I learn in these interviews to inform how we adapt the MCCAN model.

Topic Area 1: Demographic information on participant

- Could you describe your current role in the hospital and how you have been involved in 1) joining/remaining a MCCAN affiliate site and 2) achieving/maintaining CoC accreditation?

Topic Area 2: Theory of change

In the second part of the interview, I'd like to discuss the motivation behind joining MCCAN. I would also like to discuss the problems or barriers that exist in to providing quality cancer care or meeting CoC standards and whether and how joining and participating in MCCAN addresses those barriers. This will help me understand how and why MCCAN works.

Barriers:

- Based on your experience, can you describe barriers to providing quality cancer care or meeting CoC standards that you often see or encounter in practice? Can you describe barriers to establishing the programs and services that your cancer patient population needs?

Primary/secondary causal pathway:

- How did MCCAN help fix the barriers you just described (if at all)?
    - Probe: make sure respondent describes how MCCAN addressed barriers to change, not just which activities.

Moderators:

- Was there anything about your hospital patients, staff, or overall organization that made it easier to adopt the strategies that MCCAN offered to address the barriers?
- Was there anything about your hospital patients, staff, or overall organization that made it challenging to adopt the strategies that MCCAN offered to address the barriers?
    - Probes: patient population (% indigent, payer mix)? Care delivery? Staff buy-in?

Topic Area 3: Core functions of intervention

As I previously mentioned, we want to take the MCCAN model and adapt it so that it can be used in rural Iowa hospitals. When we're making changes to the intervention to use it in Iowa, we want to make sure we don't change an aspect of MCCAN that was critical to its success. So now I'm going to ask you to think a bit about what was driving the success of MCCAN – what was the "secret sauce"?

[Present activities from cheat sheet]

- Does this list reflect your experience with MCCAN activities? Would you omit or add any?
    - Which MCCAN activities contributed most to help you achieve accreditation?
    - Probe: What about it was essential – e.g., the person conducting the activity; mode of activity (in-person vs written)?
  - Which MCCAN activities/principles would you maintain at all costs?
  - Which MCCAN activities/principles seemed less essential, and could be cut out or modified?
